# Fine-tuning the antigen sensitivity of CAR T cells: emerging strategies and current challenges

**DOI:** 10.3389/fimmu.2023.1321596

**Published:** 2023-11-27

**Authors:** Dennis Christoph Harrer, Sin-Syue Li, Marcell Kaljanac, Markus Barden, Hong Pan, Hinrich Abken

**Affiliations:** ^1^ Deptartment of Hematology and Internal Oncology, University Hospital Regensburg, Regensburg, Germany; ^2^ Leibniz Institute for Immunotherapy, Division of Genetic Immunotherapy, Chair Genetic Immunotherapy, University Regensburg, Regensburg, Germany; ^3^ Division of Hematology, Department of Internal Medicine, National Cheng Kung University Hospital, College of Medicine, National Cheng Kung University, Tainan, Taiwan

**Keywords:** CAR, antigen downregulation, antigen escape, antigen sensitivity, targeting selectivity

## Abstract

Chimeric antigen receptor (CAR) T cells are “living drugs” that specifically recognize their target antigen through an antibody-derived binding domain resulting in T cell activation, expansion, and destruction of cognate target cells. The FDA/EMA approval of CAR T cells for the treatment of B cell malignancies established CAR T cell therapy as an emerging pillar of modern immunotherapy. However, nearly every second patient undergoing CAR T cell therapy is suffering from disease relapse within the first two years which is thought to be due to downregulation or loss of the CAR target antigen on cancer cells, along with decreased functional capacities known as T cell exhaustion. Antigen downregulation below CAR activation threshold leaves the T cell silent, rendering CAR T cell therapy ineffective. With the application of CAR T cells for the treatment of a growing number of malignant diseases, particularly solid tumors, there is a need for augmenting CAR sensitivity to target antigen present at low densities on cancer cells. Here, we discuss upcoming strategies and current challenges in designing CARs for recognition of antigen low cancer cells, aiming at augmenting sensitivity and finally therapeutic efficacy while reducing the risk of tumor relapse.

## 1 Introduction

Cellular therapy using chimeric antigen receptor (CAR) engineered T cells (CAR T cells) can induce long-lasting complete remissions in patients with advanced chemo-refractory hematological malignancies (1). The success of CAR T cell therapy was initially sparked by CAR T cell targeting CD19 expressed by B cell leukemia/lymphoma cells (2). CD19 constitutes a nearly ideal antigen as CD19 is expressed on high levels and its expression is restricted to the B cell lineage (3); CD19 CAR T cells mediated depletion of the B cell compartment can be clinically dealt by immunoglobulin substitution (4). Despite high efficacy of CAR T cells in reducing tumor load, nearly every second patient suffers from disease relapse (5) which is in most cases due to leukemic cells with downregulated or loss of CD19 resulting to antigen levels below the CAR T cell activation threshold, commonly termed as antigen escape (6). In addition, loss of CAR T cell functionality termed CAR T cell exhaustion, insufficient CAR T cell expansion, and limited CAR T cell persistence pose significant T cell intrinsic obstacles to the long-term success of CAR T cell therapy (7).

In case of CD19, a decrease in CAR cognate antigen load can be caused by various mechanisms: i) downregulated RNA expression results in reduced protein load on the cell surface which has no impact leukemic cell survival or amplification since CD19 is not vital for malignant B cells; ii) expression of CD19 splice variants affecting one of the exons 2, 4, 5, or 6, which creates loss of the targeted epitope and renders malignant cells invisible to the canonical CD19 CAR (8–10); iii) mutations in the CD19 gene which results in truncation of the extracellular CD19 domain (11, 12); iv) lineage switch from the B cell to the myeloid lineage which abrogates the expression of CD19 and other B cell associated antigens (13, 14); v) transfer of CD19 from cancer cells to T cells, called trogocytosis, which reversibly reduces the antigen load on tumor cells below CAR activation threshold (15).

The consequence of reducing the CD19 level on malignant B cells likely decides whether CAR T cell treatment will be successful since the CD19 level before treatment is crucial for durable anti-tumor response and the risk of relapse. By quantitative flow cytometry of CD19 levels in large B cell lymphoma (LBCL) cells of patients after axicabtagene ciloleucel therapy, researchers revealed that 3,000 CD19 molecules per cell at baseline is the threshold level; lower CD19 levels on tumor cells stratifies patients with high risk of relapse (5). Semiquantitative immunohistochemistry (IHC) H-score monitoring, however, was not sensitive enough to detect the threshold level.

In case of CD19 downregulation, that occurs more often than complete CD19 loss, augmenting CAR T cell sensitivity for antigen may restore recognition of target cells. In general, the term antigen sensitivity delineates the threshold of antigen required for redirected T cell activation. Two major factors govern the antigen sensitivity of CAR T cells. Firstly, the CAR components themselves determine the activation threshold through the affinity of the binding domain and through the hinge and intracellular domain facilitating downstream signaling. Secondly, the cellular signaling machinery distal the CAR itself impacts the activation threshold, such as co-stimulatory receptors, signal mediators, and transcription factors. In recent years, efforts were undertaken in this direction aiming at enhancing the sensitivity of CAR T cells for the battle against tumor cells with low antigen density.

In this review, we review how the CAR molecule and downstream signaling checkpoints can be modified to augment the recognition of tumor cells with low antigen density, and discuss the current challenges in reducing the risk of relapse after therapy.

## 2 Modifying the CAR

**Figure 1:**
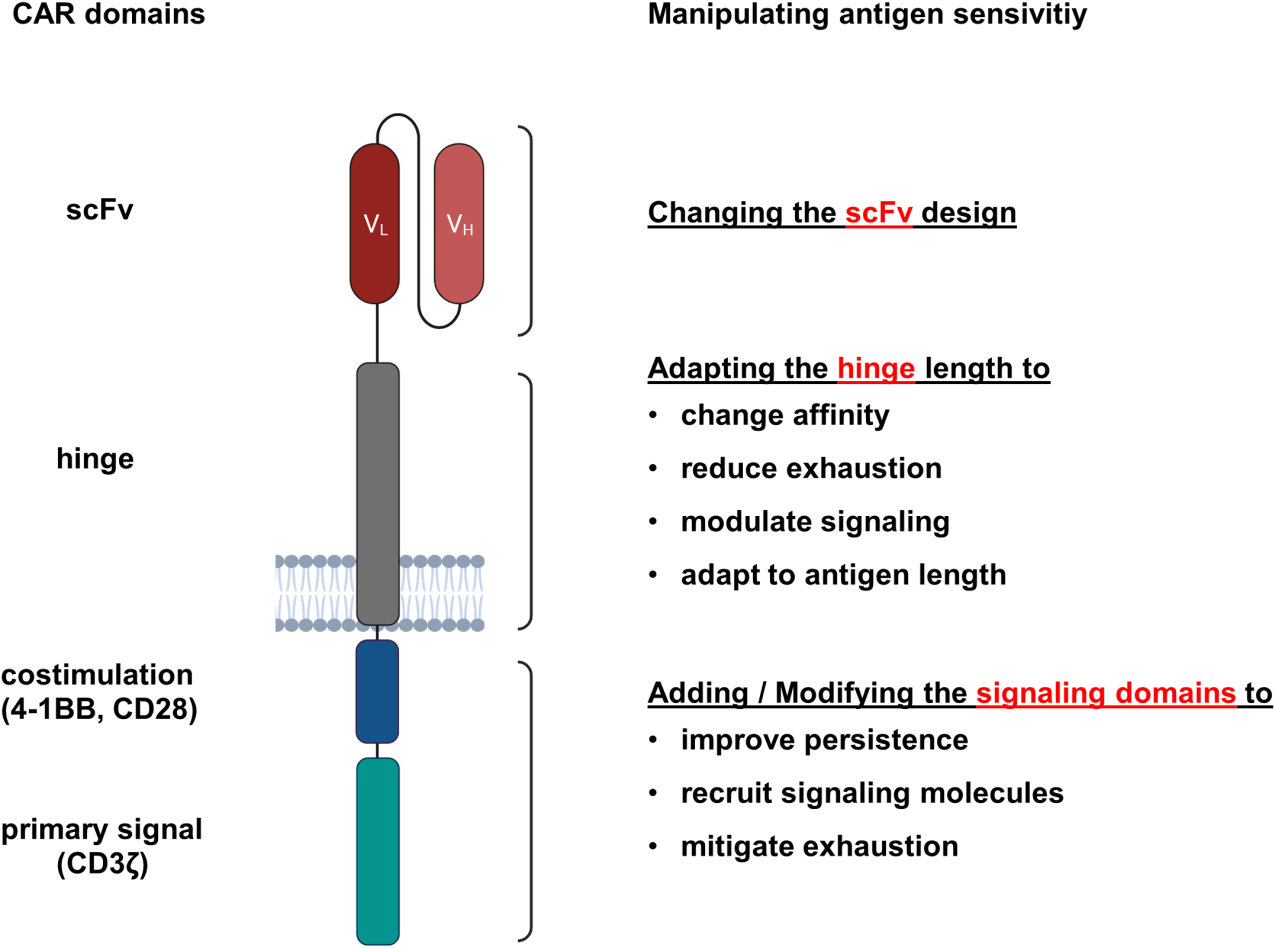
CAR domains that impact the antigen sensitivity of engineered T cells. CARs are synthetic antigen receptors consisting of an extracellular antibody-derived scFv linked via a hinge and transmembrane domain to intracellular signaling domains. For each CAR component, modifications were reported that resulted in enhanced antigen sensitivity.

The canonical “second generation” CAR recognizes target antigen by an extracellular single chain fragment of variable region (scFv) antibody that is composed of the variable domains of the antibody heavy (VH) and light (VL) chains tethered together by a short flexible linker in the order VH-VL or VL-VH (16, 17). A hinge region physically connects the scFv to the transmembrane domain and is usually derived from the IgG1 CH2-CH3 region, the extracellular part of CD28 or from the hinge domain of the CD8α molecule (18). Generally, the intracellular signaling part of a CAR comprises the CD3ζ chain to provide the primary signal and a costimulatory domain to augment and prolong activation, frequently derived of the intracellular part of CD28 or 4-1BB (18). To enhance the antigen sensitivity, each CAR component can accordingly modified (**Figure 1**).

### 2.1 Enhancing the binding affinity

The binding affinity of the antigen recognition domain substantially affects antigen sensitivity of CAR T cells (19). Accordingly, enhancing scFv affinity was investigated by various means to augment the antigen sensitivity of CAR T cells. A pioneering study nearly two decades ago using a panel of ErbB2 specific CARs that target the same epitope with different binding affinities (K_D_ 3.2 x 10^-7^ to 1.5 x 10^-11^M) revealed that the threshold for CAR T cell activation is inversely correlated with the scFv affinity (20). Correspondingly, T cells with high affinity CARs recognized targets with low ErbB2 levels, whereas CAR T cells equipped with low affinity CARs were only activated by tumor cells with high ErbB2 levels. However, the magnitude of CAR redirected T cell activation through high affinity CARs was like that of low affinity CARs when engaging ErbB2^high^ target cells. The observation was confirmed by a second study; CAR T cells with a low affinity scFv displayed equipotent anti-tumor activities against ErbB2^high^ tumors but showed reduced activation against ErbB2^low^ tumors as compared to high affinity CAR T cells (21). The concept of scFv affinity tuning for increasing antigen sensitivity was also successfully applied to CARs of other specificities, for instance GD2, EGFR, and ROR1 (22–24). In conclusion, enhancing the affinity of the scFv can basically augment antigen sensitivity of CAR T cells that, however, does not necessarily increase the strength of T cell activation.

The example of GD2-specific CAR T cells revealed the two-sided sword of affinity tuning. While CAR T cells with a 14G2a antibody derived scFv showed favorable functionality *in vitro*, no tangible anti-tumor activity was detected in murine models of neuroblastoma, which led to efforts to increase the 14G2a affinity by point mutations (22). Mutated GD2 CAR T cells showed robust activity against neuroblastoma *in vivo* but also produced severe on-target off-tumor toxicities towards the central nervous system with low GD2 levels.

To improve antigen sensitivity of ROR1 specific CAR T cells, Hudecek et al. replaced the 2A2 scFv by a high affinity scFv derived from the R12 antibody (24). High-affinity ROR1 CAR T cells evinced superior proliferation and cytokine production in response to tumor cells with low ROR1 expression as compared to 2A2 scFv based CAR T cells. In several preclinical models, no difference in CAR T cell survival was recorded for the low versus high affinity CAR T cells (24) whereas other reports suggest that low-affinity CARs are superior in boosting T cell expansion and persistence (25, 26). It is still a matter of debate whether increase of CAR affinity results in extended CAR T cell activation or in premature activation induced cell death. The consequences of increasing binding affinity on the functional T cells capacities likely need thorough exploration for each individual CAR.

To follow a more rational design, high-resolution electron microscopy images of CD19 protein interacting with the CD19 specific scFvs FMC63 and SJ25C1 guided the design of CARs with graduated antigen sensitivity (27). Those CAR T cells with increased antigen sensitivity showed improved cytotoxicity towards tumor cells with low target antigen levels. Interestingly, the different antigen sensitivities also impacted the CAR T cell susceptibility to trogocytosis upon tumor cell engagement, finally resulting in reduced antigen load on targeted cancer cells. This exemplarily demonstrates that *in silico* modelling of protein structures combined with synthetic biology can tailor the antigen sensitivity of CAR T cells to the specific requirements for detecting antigen densities found on the cancer cells.

### 2.2 Engrafting the scFv to the TCR

**Figure 2:**
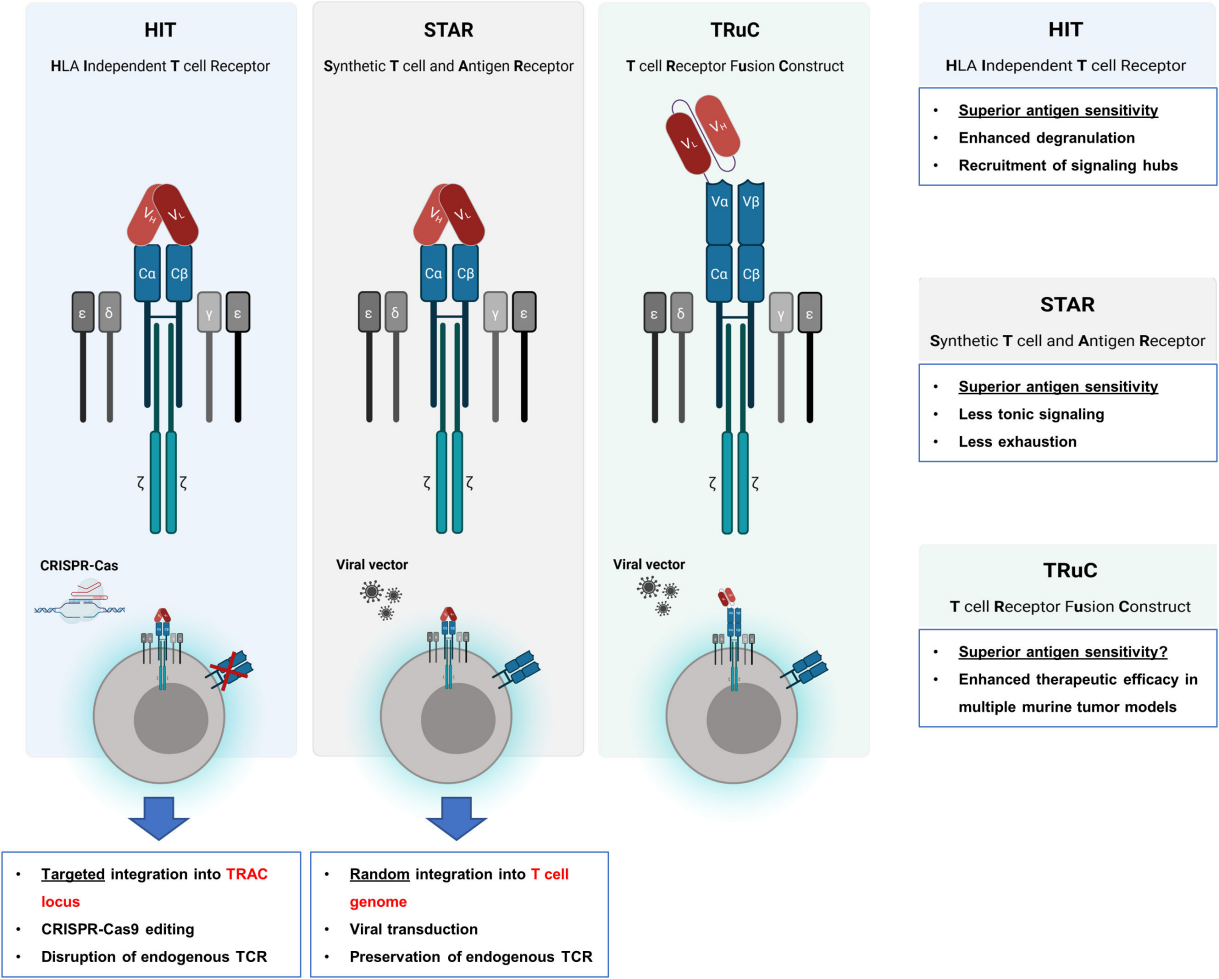
Engrafting the scFv on TCR chains. To take advantage of the superior T cell activation machinery, several strategies were reported for engrafting the scFv as binding domain to the TCR chains. By CRISPR-Cas9 genome editing the endogenous TCR chains were deleted and by AAV6 vector transfer a HLA-independent T cell (HIT) receptor was expressed that harbors the antibody VH and the VL chains linked to the TCR Cβ and Cα domain, respectively, giving rise to TCR chains with antibody derived VL and VH binding domains. Synthetic T cell and antigen receptor (STAR) is also a TCR chain-based receptor with the VH and VL chains fused to the constant regions of the TCR while the physiological TCR is still expressed. By attaching a scFv to the extracellular N-termini of the CD3ϵ chain, a synthetic TCR fusion construct (TRuC) is obtained which is effectively integrated into the physiological TCR complex. In contrast to HIT receptors, which are constructed by using genome editing to replace the variable domains of the T cell receptor by the scFv chains, STAR receptors and TRuCs are transferred to T cells by viral transduction.

There are several fundamental differences between CARs and TCRs with respect to their sensitivity to antigen. While the binding affinities of most scFvs used for CAR binding are in the nanomolar range, the antigen binding domains of physiological T cell receptors (TCRs) are in the micromolar range. However, the TCRs sense antigen in at least 100-fold lower concentration than CARs (28). Consequently, CAR T cells require 100fold higher levels of antigen on target cells than a TCR to redirect T cell activation. To merge the high antigen sensitivity of a TCR with the MHC-independent targeting of a CAR, investigators linked the scFv to the α/β chains of the TCR (**Figure 2**). By CRISPR-Cas9 mediated genome editing and AAV6 vector transfer for targeted integration, both the VH and the VL chains of a CD19 specific scFv were connected to the TCR Cβ and the Cα domain, respectively, giving rise to a synthetic receptor, termed HLA-independent T cell (HIT) receptor (29). Whereas HIT receptors and CARs proved to be equally effective in killing target cells with a high antigen density, HIT receptors showed superior elimination of target cells with low antigen load being about 10-times more sensitive to antigen than conventional CARs (29). Moreover, HIT receptors exhibit a more dynamic degranulation process against targets with low antigen densities than conventional CARs. Mechanistically, the TCR derived HIT triggers a faster remodeling of the actin cytoskeleton and a more effective deployment of lysosomes than the CAR as suggested by phosphoproteomic and morphological analyses (29). Moreover, HIT receptors are superior in displaying a more effective signal amplification after binding to antigen than CARs, which becomes obvious by the superior recruitment and activation of the linker for activation of T cells (LAT) acting as a signaling hub. The augmented antigen sensitivity of HIT receptors was further corroborated by two xenograft mouse models of leukemia using HIT T cells targeting CD19 and CD70, respectively (29). Taken together, HIT receptors display higher antigen sensitivity than conventional CARs and provide valuable tools for MHC-independent targeting of tumor cells with low antigen densities.

STAR (synthetic T cell receptor and antigen receptor) represents an alternative design of an antibody-TCR hybrid as the variable regions of immunoglobulin heavy and light chains (VH and VL) are fused to the constant regions of the T cell receptor (30). In contrast to HIT receptors, which are engineered by genome editing, the STAR is transferred to T cells by a transgenic sequence through viral transduction resulting in a STAR coexpressed with the physiological TCR on T cells (**Figure 2**). Contrary to conventional CARs, STAR receptors far less induce tonic signaling and evince little propensity to develop CAR T cell exhaustion. In addition, STAR receptors confer a higher level of antigen sensitivity and mediate T cell activation and cytolysis over a broader range of antigen densities to T cells as compared to conventional CARs. In multiple tumor models, T cells transduced with a STAR outperformed conventional CAR T cells with respect to therapeutic efficacy and functional persistence (30). By the retained endogenous TCR, the STAR is posed with the risk of mispairing with TCR chains leading to unforeseen novel specificities; the likelihood of such mispairing-based novel specificities is not studied in detail so far.

In an alternative approach, the anti-CD19 scFv was tethered to the extracellular N-terminus of a chain of the TCR complex, for instance the TCRα, TCRβ, CD3γ, CD3δ, or CD3ϵ chain, resulting in a T cell receptor fusion construct (TRuC). These fusion molecules have the advantage of being effectively integrated into the endogenous TCR complex on the T cell membrane (**Figure 2**) (31). T cells expressing a CD19 specific TRuC eliminated tumor cells in an antigen specific fashion and showed superior therapeutic efficacy in mice bearing CD19^+^ lymphoma cells as compared to canonical second generation CARs with either CD28 or 4-1BB costimulation (31). In comparison to HIT receptors, TRuCs may also be superior to conventional CARs in targeting tumor cells with low antigen density which, however, remains to be addressed.

To uncover the mechanism underlying the enhanced antigen sensitivity of TCR-scFv fusion components, Burton et al. systematically investigated the impact of T cell accessory receptors on antigen sensitivity. The much higher antigen sensitivity of TCRs compared to CARs originated from the insufficiency of CARs to engage accessory receptors, such as CD2 and LFA-1 (32). In line with this conclusion, the enhanced antigen sensitivity of HIT receptors, STARs, and TRuCs coincided with the enhanced capacity of those receptors to engage CD2 (32) pointing to the impact of the accessory molecules in facilitating signaling through the TCR downstream signaling machinery.

### 2.3 The impact of the hinge and transmembrane domain

Apart from the CAR binding domain, the hinge domain between the scFv and the intracellular domains impacts the antigen sensitivity. Seeking to improve the *in vivo* efficacy of a CAR in targeting CD70 on myeloid blasts and to obviate cleavage of the CD27 based CAR hinge, Leik et al. applied an *in silico* approach to identify putative metalloprotease cleavage sites within the hinge region. As a result, the hinge and transmembrane domain was substituted by the respective parts of the CD8α chain (33). As assessed by acoustic force microfluidic microscopy evaluating the cellular binding avidity between the CD70 specific CAR T cells and AML tumor cells, CD70 specific CARs with the CD8α hinge conferred augmented binding avidity on T cells as compared to CD27 hinge CARs. Mechanistically, the novel CAR design entailed a higher resistance to proteolytic cleave. Finally, the improved antigen sensitivity of CD70 specific CAR T cells eventuated in an enhanced therapeutic efficacy in a stress model of acute myeloid leukemia (33).

To define the antigen sensitivity of the FDA approved CD19 CAR constructs, Majzner et al. identified the CD28 derived hinge of axicabtagene ciloleucel being crucial for the superior responsiveness to tumor cells with low antigen density compared with tisagenlecleucel, that has the CD8α as hinge and transmembrane domain (34). The CD28 derived hinge imparted a higher antigen sensitivity, irrespectively of CD28 or 4-1BB costimulation. Accordingly, CAR T cells with a CD28 hinge displayed superior cytotoxicity against CD19^low^ leukemia cells *in vitro* and in a mouse model compared with CD8α hinge CAR T cells (34). The same observations were made with HER2 specific CAR T cells in a xenograft mouse model of HER2^low^ osteosarcoma, with B7-H3 specific CAR T cells in a model of B7-H3^low^ neuroblastoma, and with glypican-3 specific CAR T cells in a model of glypican-3^low^ neuroblastoma (34). The effect was also seen in first generation CARs without costimulation. Here, the CD28 derived hinge also boosted antigen sensitivity and overall functionality (34). Mechanistically, the downstream mediator ZAP70 is more efficiently recruited by CARs with the CD28 hinge which results in a faster orchestration of the synapse formation as revealed by total internal reflection fluorescence (TIRF) microscopy (34).

In another study, Casucci and colleagues incorporated a novel extracellular spacer based on the low-affinity nerve-growth-factor receptor (NGFR) into the CAR design (35). After screening a panel of four different spacer designs, optimized NGFR-spaced CAR T cells targeting various antigens, such as CD44 isoform variant 6 (CD44v6), CEA, and CD19, were generated. Importantly, those NGFR-spaced CAR T cells could be efficiently positively isolated using clinical-grade immuno-magnetic beads without impairing CAR T cell functionality. As an additional benefit, NGFR-spaced CAR T cells, which proved to be highly functional in animal tumor models, could be tracked with antibodies directed against NGFR (35).

Seeking to improve the antigen sensitivity of ROR1 specific CAR T cells with CD28 costimulation, Hudecek et al. truncated the IgG4 hinge-CH2-CH3 to obtain an intermediate hinge-CH3 and a hinge-only version. Using various ROR1^+^ tumor cell lines as target cells, the short version of the hinge domain conferred the highest cytotoxicity and the greatest proliferative capacity on CAR T cells (24). This is likely due to optimizing the synaptic gap between CAR T cells und target cells, which is usually around 10-30 nm for TCR/MHC recognition (36). Adjusting the length of the extracellular hinge region to optimize CAR T cell activation was reported for several other targets as well (37), supporting the general rule that membrane distal target epitopes need a short linker, membrane proximal targets a long linker for sensing low antigen levels, and finally efficient T cell activation. On the other hand, the enhanced antigen recognition of ROR1 specific CAR T cells with a short hinge resulted in increased activation induced cell death (AICD) (24) pointing to the observation that strong activation often produces short-lived CAR T cells. Collectively, the hinge between scFv and intracellular domains poses an avenue to enhance the antigen sensitivity of CAR T cells which, as a caveat, may also result in augmented AICD.

### 2.4 Engineering the signaling domain

**Figure 3:**
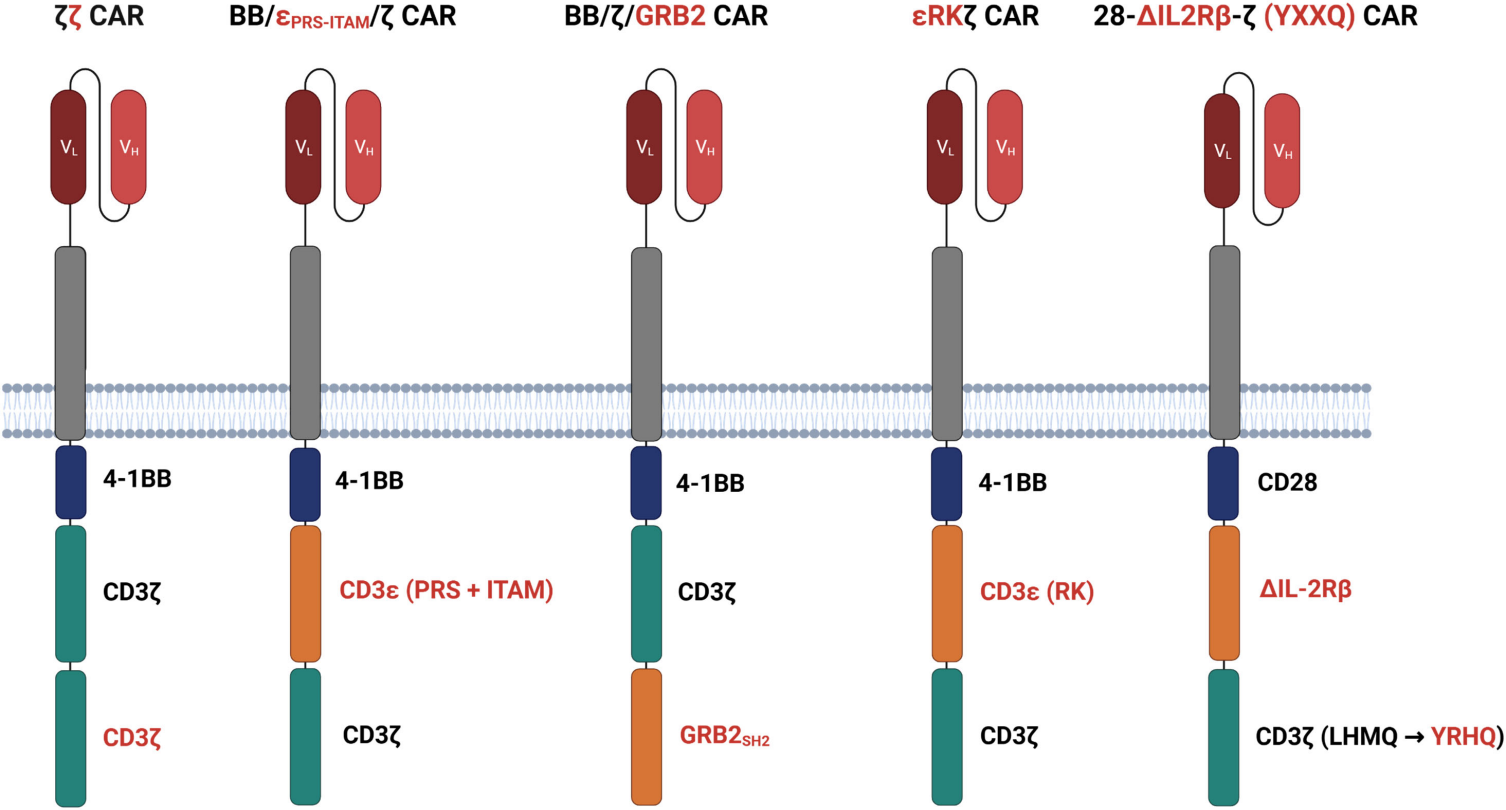
Modifying the signaling domains. ζζ CARs harbor an additional CD3ζ domain to enhance the antigen sensitivity of CAR T cells with 4-1BB costimulation. BB/ϵ_PRS-ITAM_/ζ CARs incorporate an ITAM and a CD3ϵ derived proline-rich sequence (PRS) between the 4-1BB and CD3ζ domain. In BB/ζ/GRP2 CARs, the adaptor molecules growth factor receptor bound protein-2 (GRB2) is interposed between 4-1BB and the CD3ζ domain. Both adding CD3ϵ and or GRB2 increase calcium mobilization and LAT recruitment to the signaling moieties as compared to conventional CARs. ϵRKζ CARs feature the LCK binding motif of the CD3ϵ chain, termed receptor kinase (RK) motif, between the 4-1BB domain and the CD3ζ chain improving LCK recruitment to the CAR and finally T cell activation. 28-ΔIL2RB-z (YXXQ) CARs harbor a truncated cytoplasmic IL-2Rβ domain between CD28 and CD3ζ and a STAT3-binding YXXQ motif within the CD3ζ chain, both resulting in activation of the JAK-STAT3/5 pathway.

The primary signal of most CARs is provided by the intracellular CD3ζ chain encompassing three immunoreceptor tyrosine-based activation motifs (ITAMs) which are phosphorylated upon antigen binding to initiate T cell activation (38). To investigate whether the addition of further ITAMs furthermore enhances the antigen sensitivity of CAR T cells, a 4-1BB costimulatory CAR was engineered with two CD3ζ chains (ζζ), making up six ITAMs in total (34) (**Figure 3**). Upon antigen stimulation, a CAR with two CD3ζ chains mediated stronger T cell activation at lower antigen concentrations compared with the conventional CAR with one CD3ζ chain (34). Noteworthy, tonic signaling did not increase. In a B cell leukemia model with engineered low CD19 levels, ζζ CAR T cells imposed superior tumor control than conventional CAR T cells. These data corroborate the conclusion that strengthening the primary activation signal by adding additional ITAMs into the CAR signaling backbone increases antigen sensitivity against tumor cells with low antigen load. It is not clear, whether the conclusion also holds for CD28 costimulated CAR T cells as canonical CD28 CAR T cells display higher IL-2 production and tumor control in response to CD19^low^ leukemia compared to 4-1BB-costimulated ζζ CAR T cells (34).

While adding additional ITAMs for primary activation improves the CAR T cell response, CARs with only the proximal ITAM experience less exhaustion, exhibit longer persistence, and impose greater control against tumor cells with a high antigen load (39). In contrast, CAR T cells with only one ITAM displayed a lower antigen sensitivity as compared to conventional CAR T cells (34). A caveat of adding additional ITAMs is therefore the risk of excessive CAR signaling and ensuing T cell exhaustion when engaging cells with high antigen load.

The antigen sensitivity of CAR versus TCR T cells was compared by Salter et al. who generated bispecific T cells simultaneously recognizing the Epstein-Barr virus (EBV) epitope RAK through the endogenous TCR and the tumor antigen ROR1 through a CD28 costimulated CAR (28). Consistent with previous reports, TCR outperformed the CAR in T cell activation through low antigen densities (28). Phosphoproteomic analysis revealed that CD3σ, CD3ϵ, and LAT were more intensely phosphorylated upon TCR than CAR activation. 4-1BB CAR T cells showed an even less pronounced CD3σ, CD3ϵ, and LAT phosphorylation as compared to CD28 costimulated CAR T cells (28). Driven by the hypothesis that insufficient CD3σ and CD3ϵ phosphorylation restricts LAT recruitment and subsequent CAR T cell activation, the authors inserted both the ITAM and proline-rich sequence (PRS) of the CD3ϵ chain between the 4-1BB and CD3ζ signaling chains (**Figure 3**). Alternatively, the growth factor receptor bound protein-2 (GRB2) was interposed between 4-1BB and CD3ζ as adaptor molecule (**Figure 3**). Both CD3ϵ and GRB2 added CARs increased calcium mobilization and led to improved LAT recruitment as compared with conventional 4-1BB CAR T cells (28). Accordingly, CARs with additional CD3ϵ or GRB2 redirected T cells more efficiently against ROR1^low^ tumors in a mouse model extending survival of tumor bearing mice compared with the canonical 4-1BB CAR T cells. The observation was also corroborated in mouse models of lymphoma and leukemia (28).

Having identified a previously unrecognized binding motif for LCK in the proline-rich sequence of the CD3ϵ chain, termed receptor kinase (RK) motif, Hartl et al. designed the strategy to amplify CAR signaling by inserting the RK motif between the 4-1BB and the CD3ζ signaling chain (ϵRKζCAR) (40) (**Figure 3**). The RK motif improved association of LCK with the CAR and facilitated CAR T cell activation. Under conditions of decrementing CAR T cell doses in leukemia bearing mice, ϵRKζ CAR T cells still imposed tumor control where conventional CAR T cells did not, resulting in a marked increase in survival. The authors concluded that the RK motif within the CAR can amplify redirected T cell activation, most likely due to increased LCK recruitment (40). It was not addressed whether the enhanced signaling through the ϵRKζ CAR also translates into an improved recognition of antigen^low^ target cells.

Apart from components of the CD3 complex, signaling motifs derived from cytokine receptors were also successfully integrated into the CAR backbone to expedite signal generation upon CAR binding (41). In this design, a truncated cytoplasmic domain of IL-2Rβ chain was inserted between the CD28 domain and the CD3ζ chain that was additionally modified to include the STAT3 binding YXXQ motif (**Figure 3**). Upon antigen encounter, the ΔIL2RB-z (YXXQ) CAR mediated activation of the JAK-STAT3/5 pathway resulting in superior therapeutic efficacy in multiple tumor models and outperforming conventional CD28 and 4-1BB CAR T cells (41). Similar to ϵRKζCAR T cells, 28-ΔIL2RB-z (YXXQ) CAR T cells may also show an improved recognition of antigen^low^ tumor cells which, however, is waiting for experimental proof.

## 3 Engineering signaling checkpoints

After antigen triggered CAR signaling, an intricate network of downstream checkpoints, including the MAPK/ERK pathway and associated transcription factors, governs the strength and duration of CAR T cell activation (42, 43). To modulate antigen sensitivity, the network itself may be a valuable target since it impacts the fate of T cell activation. In a systematic genome-wide CRISPR gene silencing approach carried out under immunosuppressive conditions, Carnevale et al. identified the RAS GTPase-activating protein RASA2 as a crucial checkpoint responsible for the acquisition of traits associated with T cell dysfunction (44). Correspondingly, targeted deletion of RASA2 in CAR T cells augmented signaling through the MAPK pathway resulting in enhanced cytotoxicity, cytokine secretion, proliferation, metabolic capacities, and superior tumor control in xenograft models as compared to non-modified CAR T cells. Noteworthy, the antigen sensitivity of RASA2^ko^ CAR T cells was substantially improved as reflected by targeting of NALM6 leukemia cells with low antigen load. The increase in antigen sensitivity was CAR independent as these cells evinced higher levels of MAPK pathway activity and proliferation upon low dose TCR/CD28 stimulation (44). Taken together, accumulating data indicate that T cell tuning by deleting the inhibitory signaling checkpoint RASA2 can augment the antigen sensitivity of CAR T cells (45).

Seeking to refine the antigen sensitivity of glypican-2 (GPC2)-specific CAR T cells, which failed to control the growth of GPC2^+^ neuroblastoma cells, Heizeneder et al. demonstrated that transgenic expression of the transcription factor c-Jun augmented antigen sensitivity of CAR T cells and enhanced their therapeutic efficacy against GPC2^low^ neuroblastoma *in vivo* (46). Moreover, in mice simultaneously engrafted with GPC2^high^ and GPC2^low^ neuroblastoma cells with 10,000 versus 5,000 GPC2 molecules per cell, CAR T cells coexpressing c-Jun completely eradicated neuroblastoma cells in a lasting fashion whilst canonical CAR T cells targeted GPC2^high^ neuroblastoma cells while leaving GPC2^low^ cells untouched (46). Given the low GPC2 expression by health tissues, on-target off-tumor toxicity by CAR T cells with engineered high antigen sensitivity is a clinically crucial issue. However, no on-target off-tumor toxicity by c-Jun overexpressing GPC2 CAR T cells was recorded (46).

c-Jun overexpression in CAR T cells can also counteract T cell exhaustion and increase antigen sensitivity by virtue of direct transcriptional activation of key genes linked to T cell functionality, such as IL-2 (47). Mechanistically, c-Jun overexpression eventuates in a displacement of AP1-IRF4 complexes from chromatin which results in impaired transcriptional programs linked to T cell dysfunctionality and in turn in increased antigen sensitivity of CAR T cells (47).

Recently, we reported that downregulation of the transcription factor interferon regulatory factor-4 (IRF4) improved antigen sensitivity which enabled CAR T cells to target antigen^low^ pancreatic cancer cells that are not recognized by CAR T cells with physiological IRF4 levels (48). IRF4 downregulation provoked an overall increase in T cell activation in response to antigen^low^ pancreatic cancer cells reflected by the upregulation of members of the IL-2 signaling pathway, such as CD25 and phospho-STAT5 (48). We hypothesized that a direct increase in c-Jun-mediated transcriptional activation of target genes, such as IL-2, through displacing AP1-IRF4 complexes from chromatin is likely the driving factor, similar to the effects obtained by c-Jun overexpression. Thus, the expedited transcriptional access to essential T cell effector genes, like IL-2, may rationalize the increased sensitivity to antigen. As IRF4 constitutes an inhibitory signaling checkpoint blunting the antigen sensitivity of CAR T cells, downregulating IRF4 expression provides increased sensitivity to antigen^low^ target cells.

## 4 Current challenges in CAR T cell therapy with improved antigen sensitivity

Cancer cells often escape CAR T cell attack during therapy by decreasing the level of target antigen rendering themselves invisible to CAR T cells and capable to allow tumor relapse. While in this situation increasing CAR T cell sensitivity to target antigen is clinically demanded, several strategies are currently evaluated comprising both modifications of the CAR molecule itself and of intracellular signaling checkpoints. No CAR construct with engineered augmented antigen sensitivity has entered clinical evaluation so far.

There are several hurdles that make increased antigen sensitivity a double-sided sword. Since most targeted antigens are also physiologically expressed by healthy cells, although at lower levels, increasing CAR T cell sensitivity in antigen recognition increases the risk for on-target off-tumor toxicities against healthy tissues. In order to enhance safety when targeting tumor antigens with co-expression in non-malignant tissue, logic-gate engineered CAR T cells and cooperative CAR targeting emerged as possible future solutions (49–51). Nevertheless, an ideal target antigen should be tumor-selective which is rarely the case; therefore a prudent evaluation of a target antigen with respect to absence or scant expression by healthy tissues is mandatory (52). As an additional caveat for clinical application, CAR T cells with augmented antigen sensitivity are frequently kept in an elevated activation status which necessitates an increased alertness for associated side effects, such as cytokine release syndrome (CRS) (53) and immune effector cell-associated neurotoxicity syndrome (ICANS) (54). In this context, the choice of a suitable target antigen with low propensity to on-target off-tumor toxicities, ideally a cancer selective antigen like mutated or onco-fetal antigens, remains the crucial safety factor for clinical application of CAR T cells with augmented antigen sensitivity.

## Author contributions

DH: writing – original draft. SL: data curation, investigation, writing – review & editing. MK: data curation, investigation, writing – review & editing. MB: data curation, investigation, writing – review & editing. HP: data curation, investigation, writing – review & editing. HA: conceptualization, supervision, writing – review & editing.
